# Rapid Evolution of Assortative Fertilization between Recently Allopatric Species of *Drosophila*

**DOI:** 10.1155/2012/285468

**Published:** 2012-01-18

**Authors:** Yasir H. Ahmed-Braimah, Bryant F. McAllister

**Affiliations:** ^1^Department of Biology, The University of Iowa, Iowa City, IA 52242, USA; ^2^Department of Biology, University of Rochester, Rochester, NY 14627-0211, USA

## Abstract

The virilis group of *Drosophila *represents a relatively unexplored but potentially useful model to investigate the genetics of speciation. Good resolution of phylogenetic relationships and the ability to obtain fertile hybrid offspring make the group especially promising for analysis of genetic changes underlying reproductive isolation separate from hybrid sterility and inviability. Phylogenetic analyses reveal a close relationship between the sister species, *Drosophila americana *and *D. novamexicana*, yet excepting their contemporary allopatric distributions, factors that contribute to reproductive isolation between this species pair remain uncharacterized. A previous report has shown reduced progeny numbers in laboratory crosses between the two species, especially when female *D. novamexicana *are crossed with male *D. americana*. We show that the hatch rate of eggs produced from heterospecific matings is reduced relative to conspecific matings. Failure of eggs to hatch, and consequent reduction in hybrid progeny number, is caused by low fertilization success of heterospecific sperm, thus representing a postmating, prezygotic incompatibility. Following insemination, storage and motility of heterospecific sperm is visibly compromised in female *D. novamexicana*. Our results provide evidence for a mechanism of reproductive isolation that is seldom reported for *Drosophila *species, and indicate the rapid evolution of postmating, prezygotic reproductive barriers in allopatry.

## 1. Introduction

One of the main goals in recent studies of speciation has been to identify the underlying genetic components of reproductive isolation and the evolutionary forces that cause their divergence [1]. Studies of speciation in *Drosophila*, with particular emphasis on the *melanogaster *and *pseudoobscura* species complexes, have begun to reveal the genetics of postzygotic reproductive barriers such as hybrid sterility and hybrid inviability [2–6]. Other forms of reproductive isolation, such as those acting after copulation and before successful fertilization of the egg, have received less attention in studies of *Drosophila* species. The incidence of this form of isolating barrier is hitherto unknown in the genus *Drosophila*; however, accumulating evidence indicates that postmating, prezygotic isolation is strong in select subgroups, such as the *virilis *group [7, 8] where postzygotic barriers are weak.

The ability to obtain fertile hybrid progeny from laboratory crosses between widely divergent species makes the *virilis* species group a particularly good system to investigate mechanisms of reproductive isolation other than hybrid sterility and inviability [7, 9–11]. Informed by molecular phylogenetic analyses of species within the virilis group [12–14], speciation studies have the potential to elucidate the temporal accumulation of reproductive incompatibilities associated with increased divergence. The closely related species pair *D. americana* and *D. novamexicana* is particularly relevant to the study of the initial incompatibilities that accumulate following geographic isolation. Contemporary geographic distributions of these species are separated, respectively, east and west of the Rocky Mountain Range, with isolation estimated to have occurred ~0.4 mya [14]. These allopatric distributions were established in North America following colonization from Eurasia, where a much older (4.5 mya) divergence occurred with *D. virilis*. Previous studies have primarily investigated reproductive incompatibilities involving *D. virilis* [8, 15, 16], while incompatibilities between the more recently diverged species pair, *D. americana* and *D. novamexicana*, have not been studied beyond the early experiments of Patterson and Stone [17].

While *D. americana* and *D. novamexicana* exhibit clear morphological divergence [18–20], studies of genetic differentiation reveal conflicting patterns depending on the genomic regions examined [14, 17, 21–25]. At the chromosomal level, the species differ by several rearrangements. A fixed centromeric fusion between two autosomes (chromosomes 2 and 3) is present in *D. americana*. Another centromeric fusion between the X and 4th chromosomes is also unique to *D. americana*, but this rearrangement still segregates with the ancestral chromosome forms and exhibits a strong latitudinal cline [26, 27]. Historically, this rearrangement has been used to differentiate northern and southern subspecies of *D. americana *[11, 28]. In contrast to the ancestral arrangement of chromosome arms maintained by *D. novamexicana*, several derived inversions have been fixed, yet in each case both the ancestral and derived arrangements continue to segregate in *D. americana* [29]. These chromosomal rearrangements with their associated sequence variants have the potential to influence patterns of genetic differentiation locally throughout the genomes of these species.

 Genetic diversity and differentiation of these sister species appear to be influenced by their distinct demographic histories and the sorting of ancestral variation during the short time since their isolation. Both species occupy riparian habitats; however, *D. americana* is broadly distributed in the mesic environs of the central and eastern USA, whereas *D. novamexicana* is only known from isolated localities in the xeric environs of the southwest USA [11]. Different effective population sizes are evident from the high sequence diversities maintained within nuclear and mitochondrial genomes of *D. americana* compared to the essentially invariant *D. novamexicana* [14, 22, 23, 25]. Phylogenetic analyses of individual loci mostly recover single, but sometimes multiple, monophyletic groups of alleles from *D. novamexicana* embedded within diverse alleles of *D. americana*. This paraphyly indicates that a large portion of the variation currently segregating in *D. americana* is ancestral to the divergence between this species pair, and by contrast this ancestral variation has been either fixed or lost in *D. novamexicana*. Genomic regions are variably related between species, with shared chromosomal rearrangements having localized affects on relationships, but phylogenetic analysis of combined data from multiple genes successfully resolves reciprocally monophyletic clades of the sister species [25]. Overall, these observations are consistent with a scenario wherein, following the initial peripatric split within the *D. americana-D. novamexicana* ancestral population, a much smaller population of *D. novamexicana* persisted west of the Rocky Mountains compared with the broadly distributed populations of *D. americana* to the east.

 Given the recent divergence between *D. americana *and *D. novamexicana*, and their close genetic relationship, studies of laboratory crosses are needed to reveal barriers to reproduction that have arisen in the short interval since their isolation, thereby shedding light on the initial mechanisms contributing to the reproductive isolation between species. An investigation of mating preference among members of the virilis group by Spieth [30] suggested that behavioral isolation has not evolved between this species pair. In contrast, Patterson and Stone [10] report asymmetric reductions in the numbers of progeny produced from interspecific crosses; female *D. novamexicana* produce fewer heterospecific progeny than female *D. americana*. Here we investigate the causes of the reduced number of hybrid progeny. We specifically examine the stage of reproduction where incompatibility is expressed, and further investigate the consequences of double inseminations for conspecific and heterospecific crosses. We show that incomplete, noncompetitive gametic isolation is the primary reproductive barrier that has evolved between these species.

## 2. Methods

### 2.1. Fly Stocks and Crosses

All isofemale lines of *Drosophila americana* were derived from flies collected in 1997 or 1999 and are maintained at the University of Iowa. Last two digits of the collection year were used in the line identification. The *D. novamexicana* line (15010-1031.4 from Moab, Utah, USA) was obtained from the Drosophila Species Stock Center (Tucson, AZ). All lines were cultured on standard cornmeal medium at 22°C and with a 14 : 10 light : dark cycle.

Offspring production in conspecific and heterospecific crosses was assessed by collecting virgin flies of *D. americana* (NN97.4-red, from Niobrara, Nebraska, USA) and of *D. novamexicana* (1031.4) 24–48 hours after eclosion and aging in groups of 20–25 for 10 days. Sexually mature individual females were paired with sexually mature individual males in a yeasted food vial until mating was observed and copulation was complete. Males were aspirated from the vial and females were left to lay eggs at 22°C. Adult flies eclosed ≥21 days later and the total number of progeny produced by each female was recorded.

Double matings followed the same regime of virgin collection and aging; however, successful copulation with the first male was followed by pairing and copulation with a second male on the following day. Progeny of each doubly mated female were visibly identified as either conspecific or hybrid. In crosses involving *D. novamexicana* females, hybrids are distinctly darker in body color than conspecific offspring (i.e., due to dominance of the darker pigmentation of *D. americana*). On the other hand, hybrids produced in crosses involving *D. americana* females of the NN97.4-red line, which have a recessive eye-color mutation, exhibit wild-type eye color compared to conspecific homozygotes with mutant red eye color. These data were tested for equality in mean number of progeny using a two-sample *t*-test (JMP 8).

### 2.2. Egg Hatch in Conspecific and Heterospecific Crosses

Egg hatch in conspecific and heterospecific crosses of *D. americana* (NN97.4-red) and *D. novamexicana* (1031.4) was measured through large-scale egg collections. Males and females of the parental lines were isolated as virgins and aged 10 days. Sexually mature adults were mated in groups of 10–20 flies in yeasted cornmeal vials for 3-4 days and introduced into population cages (25 cm × 25 cm × 40 cm) at a density of approximately 200 males and 200 females per cage. Cages were supplied each day with a water source and a new grape juice agar plate containing a dollop of yeast paste. Two days after introducing flies into the cage, 100 eggs were collected daily and arrayed on a new grape juice plate over 10 days from each cross (*n* ≈ 1000 for each cross). Numbers of hatched and unhatched eggs were recorded two days after arraying each egg collection.

Variability in conspecific and heterospecific egg hatch was investigated using five additional iso-female lines (HI99.14, PM99.32, LA99.46, FP99.4, and ML97.5) of *D. americana* derived from broadly separated localities distributed throughout the Mississippi River Valley. In addition to measurements of egg hatch for each line in reciprocal heterospecific crosses with *D. novamexicana* (1031.4), conspecific crosses between lines were used to measure egg hatch between geographically separated populations of *D. americana*. Similar procedures for collecting, arraying, and determining egg hatch from population cages were used. A total of 20 heterospecific crosses between *D. americana* lines were performed. Mean heterospecific hatch rate estimates were compared to conspecific hatch rate estimates using the Tukey-Kramer HSD test (JMP 8).

### 2.3. Reproductive Incompatibility Assay

#### 2.3.1. Fertilization

The ability of conspecific and heterospecific sperm to successfully fertilize eggs was assessed using a sperm-tail specific rat polyclonal antibody, *α*-XT (provided by Tim Karr, Arizona State University), to visualize the sperm tail within eggs after laying. The two lines used for this experiment were *D. americana* NN97.4-red and *D. novamexicana* 1031.4. Eggs were collected in 30-minute intervals (to ensure eggs were obtained shortly after deposition) from conspecific and heterospecific cage populations until ≥100 were obtained for each cross. Eggs were dechorionated, fixed, and rehydrated as described in [31]. Rehydrated embryos were incubated in a 1 : 300 *α*-XT: PBTA solution for 1 hour, rinsed multiple times, and washed in PBTA overnight at 4°C. Eggs were labeled with Alexa Fluor green fluorescent anti-rat secondary antibody at a 1 : 400 dilution for 1 hour before rinsing extensively (>10 times) and washing in PBTA overnight at 4°C. The rinse/wash cycle was repeated three times. Eggs were rinsed with PBS-Azide before mounting on a slide with 90% Glycerol and observing under 40x magnifications with fluorescence illumination (Leica DM2000). The proportion of eggs containing fluorescently labeled sperm tail was recorded for each of the conspecific and heterospecific crosses. Independent samples of eggs were collected from the same population cages and arrayed on grape juice agar plates to assess the hatch rate.

#### 2.3.2. Sperm Storage and Motility

Two different experimental regimes were used to assess the efficiency of storage and motility of sperm in conspecific and heterospecific crosses involving *D. novamexicana* females, where the greatest difference in egg hatch is observed. First, *D. novamexicana* females were mated to an individual conspecific (*n* = 26) or an individual heterospecific male (*n* = 24). Subsets of females from each of the two crosses were dissected 1, 2, and 3 days after copulation. All dissections included only the reproductive organs, which were placed on a slide containing Ringer's solution and overlaid with a cover slip. Sperm motility was qualitatively assessed by phase microscopy where motile sperm appear as a bundle of colored wave-like lines.

 A second experimental strategy involved transferring *D. novamexicana* females to 2% agar immediately following mating with either conspecific or heterospecific males. The expectation is that maintenance on 2% agar would reduce the propensity for laying eggs (see Section 3). The number of eggs laid into the agar by each female was recorded after one week, when the reproductive organs were dissected and examined under a light microscope for the presence of motile sperm. Dissections in which any of the sperm storage organs were severed or ruptured were discarded. The presence or absence of sperm in either the spermathecae or seminal receptacle was recorded for each female. Motility was assigned for each of the two storage organs separately on the basis of whether the stored sperm mass displayed rhythmic oscillating motion.

## 3. Results

### 3.1. Progeny Numbers in Conspecific and Heterospecific Crosses

Consistent with results previously reported by Patterson and Stone [10], progeny numbers are reduced in interspecies crosses between *D. americana* and *D. novamexicana* relative to crosses within species (Table 1). The number of progeny produced by female *D. novamexicana* mated to heterospecific males is only about 2% of the number produced by female *D. novamexicana* mated to conspecific males (Table 1; *t* = 20.0, *d.f.* = 48, *P* < 0.001). Progeny number is not reduced as dramatically in heterospecific crosses involving female *D. americana*. In this case, female *D. americana* mated with heterospecific males produce about 30% of the progeny number of females mated with conspecific males (Table 1; *t* = 10.7, *d*.*f*. = 44, *P* < 0.001). Females of the two species produce similar progeny numbers when mated with conspecific males (*t* = −2.02, *d*.*f*. = 46, *P* > 0.05) whereas heterospecific crosses with each female *D. novamexicana* produce on average only a single individual, which is significantly less than the number produced by female *D. americana* in heterospecific crosses (*t* = −13.02, *d*.*f*. = 48, *P* < 0.05).

### 3.2. Egg Hatch in Conspecific and Heterospecific Crosses

Failure of early postcopulatory events was investigated as the cause of reduced progeny numbers in crosses between *D. americana* and *D. novamexicana*. Following collection and arraying eggs produced by females of each species, high hatch rates were observed for both species when mated with males of the same line. Hatch rate measured for eggs produced by *D. novamexicana* is 97.2% while hatch rate measured for eggs produced by *D. americana *is 89.7% (Table 1). Hatch rates for eggs produced from heterospecific crosses are reduced, consistent with the pattern observed for progeny numbers. Only ~1% of eggs produced by female *D. novamexicana* mated with *D. americana* hatch successfully (Table 1), reflecting a significant reduction in comparison with eggs produced by females mated with conspecific males (*t* = −105.6, *d*.*f*. = 18, *P* < 0.001). Hatch rate of eggs produced by female *D. americana* mated with *D. novamexicana* is 35.8% (Table 1), which is significantly reduced in comparison with the conspecific cross (*t* = 15.34, *d*.*f*. = 18, *P* < 0.001).

**Figure 1:**
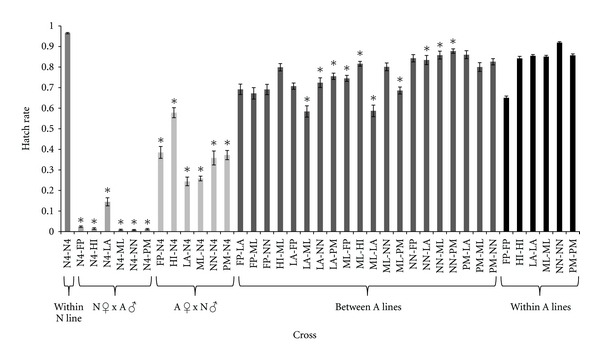
Egg hatch rate within and between *D. novamexicana* and six lines of *D. americana*(see Section 2). Egg hatch estimates “within N line” and “within A lines” are averages of within-line hatch rates from all experiments. Asterisks indicate egg hatch estimates that are significantly different between lines compared to within lines (*P* < 0.001, Tukey-Kramer HSD). A = *D. americana*, N = *D. novamexicana*. N4 is the *D. novamexicana* line used throughout the study (1031.4). All remaining two-letter abbreviations are the first two letters of each *D. americana* iso-female line i.d. (see Section 2).

Crosses within and between multiple lines of *D. americana* reveal considerable variation in hatch rate, ranging from 65.07% to 91.85% for crosses within lines and from 58.4% to 87.75% for crosses between lines (Figure 1). Overall, the average hatch rate for eggs produced from matings within lines is not significantly different from the average hatch rate from matings between lines (Tukey-Kramer HSD, *P* > 0.05), although significantly reduced hatch rates were measured in crosses between several lines of *D. americana* compared to within-line controls (Figure 1). Unlike *D. americana*, hatch rate estimates between three iso-female lines of *D. novamexicana* are uniformly high with no significant difference from within-line hatch rates (data not shown).

Hatch rates were consistently reduced for eggs produced from interspecific crosses (Figure 1). Eggs of female *D. novamexicana* crossed with males from five different lines of *D. americana* exhibit the lowest hatch rates, ranging from 0.9% to 14.5%, with each significantly lower than the conspecific hatch rate within *D. novamexicana* in each respective experiment (Tukey-Kramer HSD, *P* < 0.001). In reciprocal crosses between females of the five lines of *D. americana *and male *D. novamexicana*, hatch rates range from 24.4% to 57.8%, which is significantly reduced in comparison to hatch rates within lines of *D. americana* (Tukey-Kramer HSD, *P* < 0.001). Hatch rates in the reciprocal crosses between *D. novamexicana* and *D. americana* reflect the observations from progeny numbers; reductions in egg hatch and adult progeny are greatest in heterospecific crosses involving female *D. novamexicana*, and less severe in heterospecific crosses involving female *D. americana*.

### 3.3. Reproductive Incompatibility

**Figure 2:**
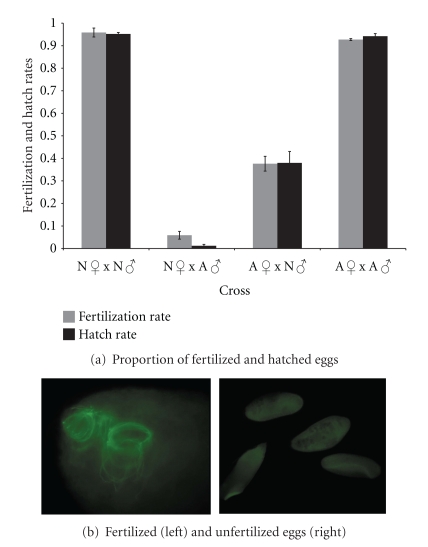
(a) Proportion of fertilized and hatched eggs (error bars represent standard error). (b) Egg containing fluorescently labeled sperm tail (left) and eggs containing no sperm tail (right).

Reduced egg hatch in heterospecific crosses may be due to postcopulatory problems arising before fertilization, during fertilization, or early in embryogenesis. A sperm-tail specific antibody, *α*-XT, was used to visualize sperm tail in laid eggs to determine whether they were successfully fertilized. Eggs were classified as fertilized if the sperm tail was clearly visible in the anterior part of the egg (Figure 2(b)). No case of partial or incomplete fertilization was observed as has been previously described in crosses between two races of *D. melanogaster *[32]. The results, shown in Figure 2(a) alongside hatch rate measurements from eggs produced from the same set of crosses, indicate a direct correspondence between the proportion of fertilized eggs and the proportion of hatched eggs in all crosses. Although the hatch rate of eggs laid by *D. novamexicana* females mated to *D. americana* males is slightly higher than the fertilization rate, indicating possible additional incompatibilities early in development, this difference is not significant (*z* = 0.397, *d*.*f*. = 1, *P* > 0.05). No difference is observed between hatch rate and fertilization rate in the other three crosses (*z* ≈ 0, *d*.*f*. = 1, *P* > 0.05). This result indicates that the failure of heterospecific sperm to fertilize is the main, if not the only, contributor to reduced hybrid production and suggests minimal postzygotic incompatibility in early embryogenesis.

The reduced capacity for heterospecific sperm to successfully fertilize may arise during storage in the female reproductive tract, or may reflect the heterospecific sperm's inability to penetrate the egg. To assess the former, we investigated sperm motility and storage dynamics in both conspecific and heterospecific inseminations under two experimental regimes: one in which females were provided with standard cornmeal food and yeast immediately after insemination, and another in which females were provided with only a water source (2% agar) immediately after insemination. To maximize detection of a difference in these experiments, only crosses involving *D. novamexicana* females were performed.

 In the first regime, females were dissected at four consecutive intervals after insemination (6 hrs, 24 hrs, 48 hrs, and 72 hrs after insemination). The reproductive tracts from dissected females at each time interval were observed under phase microscopy, where motile sperm appear as colored lines. This method does not provide a quantitative measurement of sperm motility, but rather a qualitative assessment of whether sperm, if present, are motile or not. The pattern of sperm motility within storage organs was indistinguishable between conspecific and heterospecific inseminations up to three days after insemination (results not shown).

 To further investigate the dynamics of sperm storage and motility within the female reproductive tract, we employed a strategy in which we kept inseminated females in suboptimal ovipositing media, namely, 2% agar. The goal here is to reduce sperm utilization by reducing oviposition propensity and to assess whether a difference in storage and motility is detectable between conspecific and heterospecific inseminations following a prolonged period of storage. To validate that egg-laying was reduced by maintaining inseminated females on 2% agar, we divided the two classes of inseminated females (conspecific and heterospecific) into two rearing conditions after insemination: a subset of each class was placed in standard cornmeal, yeasted media, and another subset on 2% agar (1 female/vial). We monitored the number of eggs laid in each vial; all inseminated females reared in standard media laid a large number of eggs, whereas half of all inseminated females reared on 2% agar laid eggs, 31 being the highest number of eggs laid, which is less than half of what would be conservatively expected under optimum conditions (*≈*71 eggs/insemination, calculated from Table 1). This suggests that rearing inseminated females on 2% agar reduces oviposition, and potentially prolongs sperm storage.

**Figure 3:**
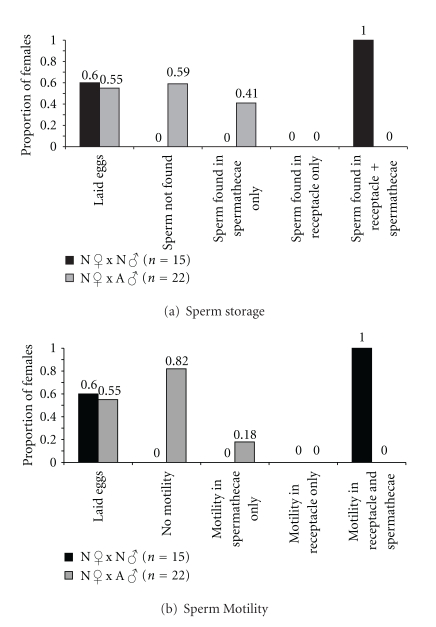
Proportion of inseminated females reared in 2% agar that contained (a) stored sperm and (b) had motile sperm. The left-most column in both (a) and (b) shows the proportion of conspecifically and heterospecifically mated females that laid eggs in 2% agar.

 For each inseminated female reared for 7 days on 2% agar, the intact ventral receptacle and the two spermathecae were dissected and separately classified according to whether they contained sperm, and whether the sperm was motile. The results are divided into Figures 3(a) and 3(b) representing sperm storage and sperm motility, respectively. In conspecific inseminations, sperm was found in all cases to be stored in both storage organs, whereas 41% of heterospecifically inseminated females contained sperm only in the spermathecae (Figure 3(a)) (*χ* ^2^ = 13.66, *d*.*f*. = 1, *P* < 0.001).  Figure 3(b) shows the motility status of the stored sperm: in conspecific inseminations, motile sperm was detected in both storage organs in all inseminated females, whereas only 18% of heterospecifically inseminated females contained motile sperm, which was found only in their spermathecae (*χ* ^2^ = 23.90, *d*.*f*. = 1, *P* < 0.001). These results clearly indicate that there is substantial loss of sperm in the heterospecific cross, particularly from the seminal receptacle. The low percentage of motile sperm that is found only in the spermathecae suggests that sperm may be further incapacitated or rendered inviable after prolonged storage in this organ.

### 3.4. Progeny Numbers in Double Matings

**Figure 4:**
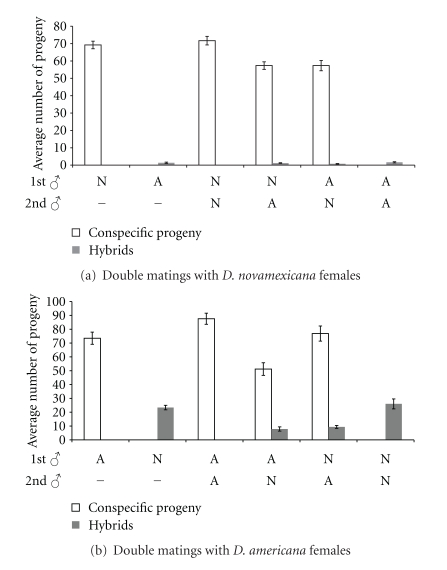
Average number of progeny produced by (a) *D. novamexicana* and (b) *D. americana* females when singly and doubly mated to conspecific and heterospecific males (error bars represent standard error).

Double mating experiments were designed to examine two postcopulatory phenomena: (1) whether the ejaculate of heterospecific males incapacitates the female reproductive tract or otherwise directly reduces the reproductive success of conspecific sperm and (2) whether the presence of the conspecific ejaculate influences the reproductive success of heterospecific sperm. The results for single and double matings are summarized in Figure 4.

First, a comparison between conspecific crosses involving a single male and double matings involving a heterospecific male reveals a modest, yet significant reduction in conspecific progeny numbers produced by female *D. novamexicana* mated with males of both species irrespective of mating order (Figure 4(a); N-A: *t* = 4.26, *d.f.* = 48, *P* < 0.05. A-N: *t* = 4.12, *d.f.* = 45, *P* < 0.05.). The consistent reduction in conspecific progeny suggests that the *D. americana* ejaculate partially incapacitates the female *D. novamexicana* reproductive tract and/or directly interferes with conspecific sperm. Conversely, fewer conspecific progeny are produced in double matings involving female *D. americana* only when *D. novamexicana* is the second copulating male (*t* = 4.11, *d.f.* = 37, *P* < 0.05), whereas conspecific progeny numbers are not reduced when *D. novamexicana* copulates first (Figure 1(b): *t* = 0.61, *d.f.* = 36, *P* > 0.05). The ejaculate of *D. novamexicana* either interferes with or displaces resident conspecific sperm in the female reproductive tract of *D. americana*, but the female reproductive tract is not incapacitated since similar progeny numbers are produced from single matings with male *D. americana* and from double matings where *D. novamexicana* copulates first.

 Second, we investigated whether conspecific sperm precedence (CSP) is operating in double inseminations. The main indication of CSP is a reduction in the number of hybrid progeny when a female is inseminated by a heterospecific and conspecific male relative to a female singly inseminated by a heterospecific male. Both orders of double matings involving female *D. novamexicana *produce an equal number of hybrid progeny as the single heterospecific cross (N-A: *t* = 0.47, *d.f.* = 48, *P* > 0.05; A-N: *t* = 1.67, *d.f.* = 45, *P* > 0.05) (Figure 4(a)). However, since only one hybrid offspring is produced on average by each female *D. novamexicana* mated with male *D. americana*, CSP would be difficult to detect. A female *D. americana* on the other hand produce an appreciable number of hybrids when mated with *D. novamexicana*. When mated to both a conspecific and a heterospecific male, female *D. americana* produce fewer hybrids irrespective of mating order (A-N: *t* = 8.23, *d.f.*=37, *P* < 0.05; N-A: *t* = 7.302, *d.f.* = 36, *P* < 0.05) (Figure 4(b)). These results indicate that the *D. americana* ejaculate reduces the fertilization success of *D. novamexicana* sperm when both are present in the reproductive tract of female *D. americana*.

## 4. Discussion

This study reveals evidence of strong postmating, prezygotic isolation between the two closely related species *D. americana* and *D. novamexicana*. This barrier to successful fertilization following insemination appears to arise in both species from an incompatibility between the female reproductive tract and the male ejaculate of the other species. Furthermore, prolonged storage of sperm in the reproductive tract of *D. novamexicana* leads to loss and/or incapacitation of heterospecific sperm. Our method of minimizing oviposition by keeping inseminated females in 2% agar allowed us to detect differences in storage dynamics and sperm motility between conspecific and heterospecific inseminations of *D. novamexicana*. Our observations of sperm motility lack quantitative measures (which are difficult to obtain); however, we were able to detect a global difference in sperm motility between conspecific and heterospecific crosses involving *D. novamexicana*. We are unable to account for a possible difference in number of sperm transferred by males from each species during copulation; however, this difference is unlikely given that conspecific crosses in both species produce a similar number of progeny. Storage and motility dysfunctions may not be the only disruptions to successful fertilization since a direct inability of sperm to penetrate the egg cannot be ruled out. It is also possible that the recognition mechanism between sperm and egg is compromised in heterospecific crosses given that sperm is successfully stored and maintains motility for at least 72 hours after insemination in optimum ovipositing conditions (results not shown). Therefore, other possible mechanisms preventing fertilization cannot be excluded.

 Egg hatch estimates between *D. novamexicana* and all six lines of *D. americana* used in this study demonstrate that the postmating, prezygotic barrier is indeed a species-wide phenomenon. Crosses between three *D. novamexicana* iso-female lines show minimal variation in egg hatch rates (data not shown), perhaps reflecting the species' overall paucity of genetic variation. On the other hand, half of the crosses between different *D. americana* iso-female lines show significant hatch rate reductions when compared to within-line controls, although these reductions are not as dramatic as those observed between species. These within species reductions do not relate to the historically recognized subdivision between northern and southern forms of *D. americana*, but rather may reflect the high genetic variability maintained within this species. In conjunction with the pattern of genetic differentiation characterizing the two species (that alleles of *D. novamexicana* are mostly recovered as a relatively invariant subset of the larger variation segregating in *D. americana*), these observations hint at possible evolutionary dynamics causing rapid divergence of this gametic incompatibility (see below), and provide a few plausible explanations for the asymmetry in fertilization success in reciprocal heterospecific crosses. Consider, for instance, the copulatory environment encountered by females of each species in their respective ranges. *D. americana* females encounter diverse alleles from their conspecific males, and therefore require a reproductive tract that is permissive to a wide variety of genotypes in order to maximize their fertilization success. *D. novamexicana* females, on the other hand, encounter their invariant conspecifics, and therefore variation in their reproductive tract's permissiveness is unnecessary. This may explain why *D. americana* females utilize heterospecific sperm more efficiently than *D. novamexicana.* Alternatively, genetic drift may simply override selection in the smaller *D. novamexicana* population causing random loss of maternal alleles that may be more compatible with *D. americana* males. Higher genetic variation in *D. americana* may also contribute to stronger selective outcomes for males through postcopulatory sexual selection and/or genetic conflict, leading to higher divergence in paternal alleles of *D. americana* relative to *D. novamexicana*. One line of *D. americana*, LA99.46, is unique in that its hatch rate with *D. novamexicana* females is 14.5% compared to ~1% in all other *D. americana* lines examined. Lower incompatibility in this cross may be due to paternal alleles segregating in the *D. americana* population that are more compatible with *D. novamexicana* females, and possibly present at low frequency. Until the interacting genetic components in both males and females are known, the evolutionary genetic causes of reduced fertilization in reciprocal heterospecific crosses remain speculative.

 Interspecies ejaculate competition was assessed using double mating crosses, which provided insight on whether the heterospecific ejaculate plays a role in reducing reproductive success of conspecific sperm (interference), and whether the species display conspecific sperm precedence (CSP). The former may be due to either direct interference by heterospecific sperm (e.g., competition for fertilization) or by indirectly compromising the female's reproductive tract through effects imposed by the heterospecific ejaculate. We observe interference in female *D. novamexicana* irrespective of mating order, consistent with indirect interference through females. This is not observed for female *D. americana*, where conspecific progeny number is reduced only when a conspecific copulation is followed by a heterospecific copulation, possibly due to second male precedence. Further experiments are needed to elucidate the details of this phenomenon.

Double mating with female *D. americana* reveals the presence of CSP, because the number of hybrids produced is significantly decreased in the presence of the conspecific ejaculate. This phenomenon in insects (competitive gametic isolation [1]) has been observed in ground crickets [33] and other *Drosophila* species [34, 35] for which the likely mechanism is sperm displacement [36]. A recent study of storage dynamics within *D. melanogaster* females doubly inseminated by two strains differing in fluorescently labeled sperm heads reveals that second male precedence is accomplished through displacement of resident sperm [37]. In the current study the mechanism of CSP is unknown, although we expect that it may be due to the higher competitive ability of conspecific sperm. It is also likely that higher competitive ability and second male precedence jointly contribute to reduced hybrid production in doublymated *D. americana* females when *D. americana* mates second.

Gametic isolation between species may be a result of incompatibilities that evolve as a result of male-male competition and male-female coevolution within polyandrous species [38]. Sperm competition on the one hand increases male reproductive success at the expense of female fitness, to which females in turn evolve means to counteract the deleterious ejaculate effects [39–41]. This process leads to an arms-race dynamic between males and females and results in rapid evolution of interacting reproductive systems. Coevolution between the sexes also results in rapid evolution of sex-specific phenotypes, such as sperm length [42], which has been shown to correlate with higher fertilization success. In other words, these within-species dynamics result in cryptic sexual selection that likely accelerates divergence of the genes underlying sexual interactions. Seminal fluid proteins (Acps) in *Drosophila*, which facilitate many of the postcopulatory processes leading to successful fertilization (reviewed in [43]), have been shown to evolve rapidly (e.g., [44]). Our study shows that gametic incompatibility between species, which may also result from a coevolutionary dynamic within species, has arisen much more rapidly between species than within species, suggesting a role for allopatric separation in homogenizing the coevolutionary sexual interactions within species, but deeming those interactions incompatible between species. It is therefore likely that some form of cryptic sexual selection is causing the rapid divergence of traits required for proper storage, gamete recognition, and fertilization.

*D. americana* and *D. novamexicana* represent a special case in the literature because they are the only reported *Drosophila* species pair where gametic isolation is the only apparent reproductive barrier detectable in the laboratory. Given their recent divergence and allopatric distribution, they represent a unique opportunity to study the genetics of earlystage postmating, prezygotic isolation in *Drosophila*, studies that have been lacking until recently. Crosses among all four members of the *virilis* group show gametic incompatibilities with varying degrees of asymmetry and severity [7, 8], suggesting that postmating, prezygotic isolation is common and may be particularly apparent in this phylad due to the low level of postzygotic incompatibility. Genetic analysis of this form of isolation in this species group will allow us to determine whether the evolutionary dynamics characterizing postzygotic isolation (rapid evolution by positive selection) also characterize postmating, prezygotic barriers.

## Figures and Tables

**Table 1:** Progeny numbers and egg hatch rate for crosses within and between *D. americana* (NN97.4-red) and *D. novamexicana *(1031.4).

Female parent	Number of progeny (±S.E)		Egg hatch rate (±S.E.)	
	Conspecific	Heterospecific	Conspecific	Heterospecific
*D. americana*	73.5 (±0.87)	23.4 (±0.33)	0.90 (±0.009)	0.36 (±0.03)
*D. novamexicana*	69.2 (±0.44)	1.4 (±0.08)	0.97 (±0.009)	0.008 (±0.003)
